# Anaesthesia considerations and techniques for Pressurised IntraPeritoneal Aerosol Chemotherapy (PIPAC)

**DOI:** 10.1515/pp-2019-0013

**Published:** 2020-11-17

**Authors:** Venkatesan Shree, Tian Jin Lim, Lyn Li Lean, Bok Yan Jimmy So, Guowei Kim

**Affiliations:** Department of Anaesthesia, National University Health System, Singapore, Singapore; University Surgical Cluster, National University Health System, Singapore, Singapore; Yong Loo Lin School of Medicine, National University of Singapore, Singapore, Singapore; Division of Surgical Oncology, National University Cancer Institute, Singapore (NCIS), National University Health System, Singapore, Singapore

**Keywords:** Pressurised IntraPeritoneal Aerosol Chemotherapy (PIPAC) anaesthesia considerations, total intravenous anaesthesia (TIVA), anaesthesia setup

## Abstract

Pressurised IntraPeritoneal Aerosol Chemotherapy (PIPAC) is a novel surgical technique to administer aerosolized chemotherapy into the abdominal cavity as treatment for peritoneal metastasis from various cancers. As the surgery is unique and there are concerns about occupational hazards, specific anaesthetic setup and techniques are required. Notably, our institution’s experience with PIPAC has enlightened us that anaesthesia requirements during PIPAC are generally uncomplicated and that the majority of the patients undergoing PIPAC do not require invasive monitoring, advanced intra or postoperative analgesia like epidurals or PCA. The need for postoperative intensive unit care is also not required in routine PIPAC cases. We describe the anaesthetic considerations involved and the detailed preparation of staff, space, anaesthetic equipment and drugs to facilitate the appropriate modifications for anaesthesia monitoring and maintenance for an elective set up as well as our standard operating procedure for an emergency situation should it arise.

## Background

Peritoneal carcinomatosis is associated with a very poor prognosis [1] and is generally considered for palliative symptom-relieving treatment [2]. The two common types of chemotherapy used currently for palliation include systemic chemotherapy or intraperitoneal chemotherapy. The former has been found to be beneficial in the treatment of systemic metastases but not as effective against peritoneal spread due to poor blood supply to the peritoneal surface [1], [3]. Intraperitoneal chemotherapy (IPC) aims to increase the concentration of the chemotherapeutic drug while minimising systemic toxicity [1], [4]. However, IPC has limited depth of drug penetration into the tumour tissue and limited drug distribution especially in the presence of intraabdominal adhesions [5]. In addition, there are also port-related complications including infection and leakage [6].

Pressurised IntraPeritoneal Aerosol Chemotherapy (PIPAC) is a novel minimally invasive method of intraperitoneal chemotherapy to induce regression of the peritoneal metastasis in the salvage situation. Its advantage is that of higher drug concentration within the tumour cells due to pressurised vaporization. Other advantages include procedure repeatability, improved quality of life and lower morbidity due to decreased systemic toxicity from the drugs [7].

Additionally, it is useful for patients who are not eligible for cancer reduction surgery and Hyperthermic Intraperitoneal Chemotherapy (HIPEC) [2]. It is normothermic and was first introduced as a palliative treatment with tumour regression and improvement in patient quality of life [1], [7].

## The PIPAC procedure

Our institution has had experience in approximately 16 cases of PIPAC since we started PIPAC as an experimental treatment option. Patients are admitted as a same-day admission requiring a one-day stay in the hospital. A computerized tomography scan is done prior to surgery to assess the amount of disease and to plan abdominal entry. As the dose of chemotherapy required in PIPAC is 10% of the standard dose, there are minimal side effects of chemotherapy—the systemic inflammatory response symptoms and risk of procedural wound infection [1].

The PIPAC procedure as first described by Reymond et al. [1] has been performed by our team. The Medrad power injector was used with a wire running from within the operating theatre to the adjacent isolated room where it is controlled via a hand switch. Administration of the chemotherapeutic agents was via a nebulizer connected to a pressure injector via the trocar. Conditions of nebulization are: (a) application of cytostatics at a flow rate of 30 mL/min; (b) a pressure of 1380 kPa over 30 min, at room temperature (22 °C). After 30 min, the capnoperitoneum was evacuated by use of a closed-loop system through microparticle filters into the air waste system of the hospital. In our setup, we administered the cytostatics at a flow rate of 30 mL/min. We administered it for 5 min at a pressure not exceeding 200 kPa, with a total volume of 150 mL of cytostatic. After administration of the chemotherapy agents, the capnoperitoneum was evacuated via the scavenging system of the operating theatre.

All PIPAC procedures have been performed at our institute as part of an ongoing Phase I clinical trial [8], [9].

**Figure 1:**
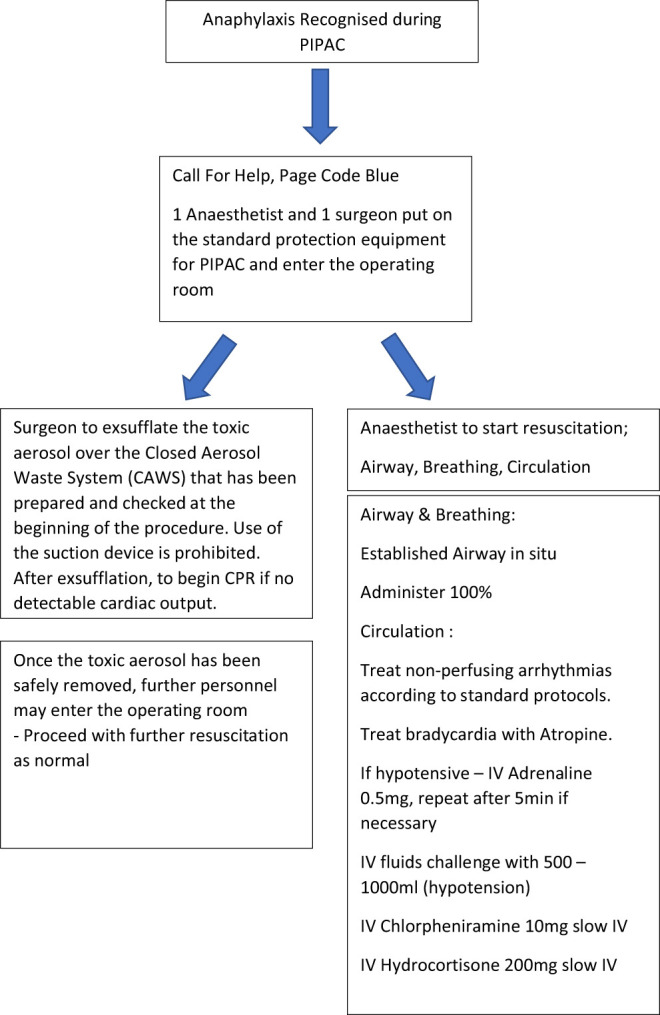
Flowchart adapted from www.Resus.org.UK [10].

## The anaesthestic considerations

In our experience, anaesthesia requirements for this procedure have been found to be relatively uncomplicated. The requirements are similar to a routine day surgery case under general anaesthesia. However, the patients undergoing this procedure may have complications associated with malignancy such as massive ascites. There may be cardiopulmonary consequences to removal of large amounts of ascitic fluid during the procedure. Patients with ascites may also warrant rapid sequence induction. Patients with poor heart or lung function pre-morbidly are also at increased risk of major adverse cardiac events. Anaesthetists should manage these cases with appropriate monitoring as required.

The preparation for this relatively short procedure is considerable and involves considerations for location, equipment and monitoring. Knowledge of these steps in preparation and optimal preoperative planning would increase theatre efficiency in a procedure that has not yet been encountered by many anaesthetic professionals.

## Location

The PIPAC procedure requires an operating room with advanced ventilation system to prevent pollution to the external environment. Additionally, an induction room enabling a closed seal to prevent potential escape of cytotoxic fumes is required. The induction room is to facilitate monitoring of the patient remotely (for staff safety), while still being ready to act in case of emergency. In an ideal set up, a monitor that depicts the patient’s vital signs both inside and outside the operating theatre (in the induction room) should be present. In a less ideal set up, the anaesthesia machine monitor swivels to face the induction room window to enable monitoring of the patient outside the theatre. In the least ideal set up, a transport monitor can be connected to the patient. We have had experience with the two latter options at our institution as we do not have a remotely located monitor in the induction room of our negative pressure operating theatre.

## Pre-operative preparation

An extensive history for pre-operative assessment should be taken from the patient to ensure that the patient meets the inclusion or exclusion criteria and to ensure patient safety.

Only routine pre-anaesthesia workup is required, and the procedure does not require any additional investigations. The patient should be counselled appropriately for a laparoscopic procedure.

The procedure is carried out under general anaesthesia. The operating theatre Closed Aerosol Waste System (CAWS) is used for the evacuation of the remnant chemotherapeutic drugs from the abdomen. Therefore, the anaesthesia machine cannot be connected with CAWS. To avoid contamination of the operating theatre with inhalational agents, a total intravenous anaesthesia (TIVA) technique should be used for induction and maintenance.

## Monitoring and equipment

Patient monitoring intraoperatively should include electrocardiograph (ECG) monitoring, pulse oximetry, capnography and blood pressure measurement. Although more intensive monitoring such as five lead ECG and intra-arterial blood pressure measurement are not required specifically for this procedure, they may be used based on the patient’s comorbid conditions.

A depth of anaesthesia monitor such as bispectral index monitoring or entropy is recommended to prevent awareness in the setting of a TIVA technique. In a similar vein, infusion extension lines using anti-reflux valves for TIVA is recommended.

## Drug administration

The PIPAC procedure is not particularly stimulating or painful and does not require use of short acting opioid infusions such as remifentanil during the procedure. Patients also do not require complex postoperative analgesia such as Epidural or PCA. We utilize a propofol TIVA technique with intermittent atracurium boluses. During PIPAC administration, it is ideal to ensure good muscle relaxation.

One intravenous line can be used for TIVA infusion while another intravenous line can be used for bolus of emergency drugs if required. Intravenous lines are extended to enable continued drug administration from a remote location outside of the operating theatre. In our institution, we generally use three 200 cm small bore extension lines for optimal extension and reduced dead space. A saline flush or infusion drip should be prepared when giving drug boluses to ensure timely delivery of drugs. Care should be taken to ensure there is no kinking of the infusion lines. The anaesthetist should check that once extended out, the infusion lines are not kinked by the closed door between the operating theatre and induction room. The TIVA pump can be either left within the operating theatre or outside the operating theatre for titration of anaesthesia. TIVA pumps and other infusion pumps should be charged continuously to make certain there are no pump failures during the procedure.

Analgesia would include simple analgesia such as paracetamol and non-steroidal agents if there are no contraindications. Infiltration of the port sites with local anaesthetic or single-shot abdominal blocks would help with pain from the port sites. In our patient population, minimal opioid usage has been required (Table 1).

**Table 1:** Standard operating procedure for PIPAC anaesthesia.

	
Location	Operating room with negative pressure/good laminar flow
	Adjacent induction room with capability to have a closed seal from operating room Power points within the room to enable charging of TIVA pumps and equipment Small window to view the patient in the operating theatre and also the monitor to view the vitals of the patient
	Ideally an adjacent room with capability to monitor the patients vitals as shown on the monitor inside in real time
Equipment	Anaesthesia machine with ventilator
	Monitor with vitals monitoring: Electrocardiograph (ECG) monitoring, pulse oximetry, capnography and blood pressure measurement Capability invasive blood pressure monitoring Bispectral index monitoring or entropy
	Monitor should be able to be swivelled towards the window of the adjacent room to enabling viewing
	TIVA pump to run Propofol infusion
	Extension lines for drug infusion
	One way valve tubing or adapter for Propofol infusion (TIVA)
Drugs	Premedication/Antiemetics:
	IV Dexamethasone (4–8 mg)
	IV Ondansetron 8 mg
	Anaesthesia drugs:
	IV Propofol for TIVA infusion
	Opioids: for intubation and intraoperative analgesia Fentanyl (0.5–1 mcg/kg) Morphine (1–2 mg as titrated, 0.1 mg/kg acute postoperative pain control) Remifentanil – is typically not required since PIPAC is not very painful
	Paralytics: IV Atracurium (0.5 mg/kg intubating dose) Suxamethonium (1–1.5 mg/kg for intubation in rapid sequence induction)
	Analgesics: Paracetamol 1 g IV (15 mg/kg dose for patients under 50 kg) NSAIDs (IV Parecoxib 40 mg. For patients <50 kg: 20 mg) Local anaesthetics
	Ropivacaine/Bupivacaine for regional anaesthesia is considered

Drug doses (reference no. [11]).

## Emergencies

Intubation to ensure a secure airway is recommended to prevent regurgitation and aspiration in the setting of laparoscopic surgery. However, a laryngeal mask airway is not absolutely contraindicated since the patients are positioned supine with no steep Trendelenburg.

The most significant adverse event that can occur is anaphylaxis due to hypersensitivity to platinum compounds used as the chemotherapeutic agent during PIPAC [12]. Although our institution uses Oxaliplatin, the risk of anaphylaxis is minimised by excluding patients with known allergy to platinum compounds. We also administer a single dose of IV steroids as part of our premedication during induction. In addition, the preparation for a potential life-threatening acute emergency such as anaphylaxis should always be considered. The drug trolley, emergency resuscitation drug trays and intravenous fluids should be available in the induction room.

Personal protective equipment should be put on if the patient needs to be acutely resuscitated or handled during the PIPAC procedure. Although correct port balloon inflation should seal the intraperitoneal chemotherapy intra-abdominally, adequate precautions should be taken in case of balloon deflation or leakage. Aerosolized chemotherapy could be cytotoxic to the lung if it escapes into the theatre environment. Recommended equipment includes a filtered mask, although barrier aprons and double gloves should be used in the unlikely instance of handling abdominal ports (Figure 1, Flow chart).

In our institution, personal protection equipment is available for both the surgeons and anaesthetists. One anaesthetist and one surgeon gowns up and enters the operating theatre for resuscitation in case of patient collapse whilst the toxic aerosol is exsufflated via the CAWS. Once the operating theatre is deemed safe and no longer cytotoxic, more personnel may enter to aid in the resuscitation.

## Conclusions

From our experience with 16 PIPAC cases, we describe the anaesthetic considerations involved and the detailed preparation of staff, space, anaesthetic equipment and drugs are required to facilitate the appropriate modifications to anaesthesia monitoring and maintenance.
